# Preosteoarthritic static posterior humeral subluxation in young adults (posterior shoulder instability type C1): associative factors, diagnosis, and treatment

**DOI:** 10.1016/j.xrrt.2025.08.009

**Published:** 2025-08-29

**Authors:** Andrea Estfeller, Alp Paksoy, Philipp Moroder, Doruk Akgün

**Affiliations:** a Medical University of Vienna, Vienna, Austria; b Charité University Hospital, Center for Musculoskeletal Surgery, Charitéplatz, Berlin, Germany; c Department for Shoulder and Elbow Surgery, Schulthess Klinik, Zurich, Switzerland

**Keywords:** Preosteoarthritic, Posterior shoulder instability, C1, Static posterior subluxation, ABC classification, SCOPE, Open wedge posterior glenoid osteotomy

## Abstract

Type C1 posterior shoulder instability represents a preosteoarthritic condition characterized by chronic, static posterior subluxation of the humeral head. Its multifactorial etiology encompasses increased glenoid retroversion, altered posterior acromial morphology, reduced humeral retrotorsion, abnormal scapulothoracic alignment, and muscular imbalances. The diagnosis relies on a comprehensive clinical history, targeted physical examination, and advanced imaging modalities that accurately detect posterior humeral head decentering, glenoid rim deficiencies, reduced posterior acromial coverage, and labral hypertrophy. Although treatment strategies range from conservative management to various surgical interventions, there has been no introduction of a procedure that reliably recenters the humeral head and halt the progression of osteoarthritis. This review aims to provide guidance in the accurate identification of C1 posterior shoulder instability by outlining its diagnostic criteria and discussing current treatment strategies.

Type C1 posterior shoulder instability (PSI), also referred to as constitutional static PSI, is a preosteoarthritic form of chronic static posterior decentration of the humeral head that predisposes young adults, particularly men, to early degenerative changes of the glenohumeral joint.^1^^,^^7^^,^^30^^,^^45^ Unlike acute posterior dislocations or dynamic subluxations, C1 PSI develops gradually over years, typically without discrete instability events, often delaying both recognition and intervention.^30^^,^^31^

Using the ABC classification, Moroder et al divided PSI into first-time dislocations (type A), dynamic subluxations (type B), and static decentering (type C), with type C further subdivided into constitutional (C1) and acquired (C2) posterior decentering.^30^^,^^31^ In C1 PSI, a constellation of anatomic factors such as increased glenoid retroversion, decreased humeral retrotorsion, reduced acromial coverage, or scapulothoracic malalignment may create a biomechanical milieu that favors posterior humeral head displacement and eccentric cartilage loading.^1^^,^^28^^,^^38^ C1 PSI may represent an early stage of primary glenohumeral osteoarthritis (OA) and could resemble the progression pattern observed in Walch B and C glenoids, *depending on the underlying osseous anatomy*.^6^^,^^45^

Although conservative and surgical options exist for C1 PSI, no consensus has emerged regarding the optimal strategy to prevent posterior humeral head subluxation and OA progression in this typically young cohort.

This review synthesizes the current evidence on type C1 PSI, covering diagnostic approaches, associative factors, and available treatment options, and highlights areas that warrant further research to optimize patient outcomes.

## Definition of C1 posterior shoulder instability

C1 PSI is defined as a chronic, static subluxation of the humeral head. It is considered congenital and atraumatic.^30^^,^^34^ This condition predominantly affects young adults, especially males, with a typical onset around the age of 30.^30^

In its early stages, C1 PSI is often asymptomatic and may be discovered incidentally. Over time, eccentric loading leads to gradual symptom development and progressive posterior cartilage damage. In more advanced stages, patients typically do not exhibit classic instability signs; instead, they report pain, weakness, and clicking sensations, likely due to posterior labral tears and cartilage degeneration resulting from eccentric wear.^30^^,^^34^

## Associative factors

Several anatomical and biomechanical factors have been implicated in the pathophysiology of type C1 PSI. Alterations in glenoid morphology, humeral torsion,^1^^,^^2^^,^^5^ and acromial morphology^4^^,^^27^ have been identified as key associative factors. In addition, functional changes, such as variations in scapulothoracic orientation and imbalances in the rotator cuff force couple, may further contribute to this condition.^1^^,^^28^

## Glenoid shape alterations

Alterations in glenoid morphology have been identified as associative factors for posterior humeral subluxation.^1^^,^^2^^,^^5^^,^^7^^,^^45^ Key alterations include an anteriorly displaced glenoid vault with an increased anterior glenoid offset and excessive glenoid retroversion.^2^^,^^30^

Recent studies have provided valuable insights into these structural variations. Akgün et al observed that shoulders C1 PSI patients, compared to healthy shoulders, show significantly increased glenoid retroversion (20.1 ± 10.7° compared with 5.8 ± 2.7° in healthy controls), along with a greater anterior glenoid offset (9.8 ± 4 mm vs. 3.9 ± 1 mm in healthy controls).^1^ Further computed tomography (CT)–based analyses have confirmed that these patients present an anteriorly displaced glenoid vault with excessive retroversion and posterior translation of the humeral head relative to the scapular blade axis.^2^ Moroder et al also reported that patients with C1 PSI exhibit the highest anterior glenoid offset (8.6 ± 5 mm) and a mean glenoid retroversion of 24.2 ± 17°, distinguishing them from other PSI subtypes.^30^

Beeler et al differentiated between static and dynamic shoulder instabilities by noting increased glenoid retroversion in Walch B1 glenoids compared to healthy shoulders. Since Walch type B1 glenoids may develop from C1 PSI, it is suggested that these anatomical variations may contribute to posterior humeral head subluxation and the subsequent development of OA.^5^

The observed structural changes, including increased retroversion and anterior glenoid offset, indicate a broader spectrum of glenoid dysplasia among PSI patients. Moroder et al found glenoid dysplasia in 45% of C1 PSI cases, with 10% showing a convex morphology,^30^ a finding confirmed by Akgün et al.^1^ These morphological variations are thought to have developmental origins and are modulated by genetic factors. Landau and Hoenecke described that the glenoid is formed by 2 separate ossification centers, with genes such as *Hoxc6* and *Emx2* regulating glenoid development.^23^ Disruptions in the ossification process may contribute to dysplastic changes seen in C1 PSI glenoids.

Moroder et al furthermore suggested that patients with an underdeveloped bony posterior glenoid rim frequently exhibit pronounced hypertrophy of the posterior labrum. These structural adaptations appear to enhance glenoid concavity, temporarily compensating for the underlying deficiency.^29^

## Humeral torsion

Humeral torsion is a critical determinant of shoulder biomechanics, influencing both joint mobility and stability.^38^ Alterations in humeral retrotorsion have been observed in patients with type C1 PSI, though it remains unclear whether these changes actively contribute to the condition's development, serve as an adaptive response, or are simply incidental.^1^

There is evidence of decreased humeral retrotorsion in individuals with C1 PSI compared to healthy controls. Initially, Walch et al described that patients with static posterior glenohumeral subluxation of the humeral head, with additional osteoarthritic changes, showed reduced humeral retrotorsion compared to normal shoulders.^45^ Akgün et al analyzed C1 PSI patients and found that they exhibited significantly lower humeral retrotorsion (14.9° ± 11.2°) compared to healthy individuals (34.4° ± 4.9°).^1^ Similarly, Raniga et al noted reduced humeral retrotorsion in Walch type B shoulders (14° ± 9°) vs. nonarthritic shoulders (36° ± 12°)^38^ and suggested that retrotorsion may remain relatively stable despite progressive OA changes. This observation raises, on one hand, the possibility that reduced humeral retrotorsion could contribute both to the initial development and the subsequent progression of posteroinferior glenoid wear, thereby influencing the observed wear pattern in these patients.^38^ On the other hand, the relationship between decreased humeral retrotorsion and increased glenoid retroversion in C1 PSI may suggest a potential compensatory mechanism, in which reduced retrotorsion may help balance the effects of a more retroverted glenoid. Supporting this, previous studies have shown that although anatomical alterations are present in C1 PSI, the glenohumeral joint appears relatively well centered when assessed using the glenohumeral subluxation index. In contrast, a clear posterior decentering is evident when evaluating alignment relative to the scapula.^1^

## Acromial morphology

Recent research has increasingly emphasized the role of acromial morphology in the pathophysiology of PSI,^1^^,^^5^^,^^17^^,^^27^ and these differences may also apply to type C1 PSI.^1^ Several studies have demonstrated that patients with PSI tend to exhibit a higher and more horizontally oriented posterior acromion compared to healthy shoulders.^1^^,^^4^^,^^16^^,^^27^ with Meyer et al noting that the posterior acromion in PSI patients is significantly higher than in those with anterior shoulder instability and that the posterior acromial tilt may be higher in the PSI group.^27^ Similarly, Beeler et al differentiated between dynamic and static forms of PSI. Although acromial morphology did not differ substantially between these subtypes, both deviated notably from normal anatomy. Specifically, patients with static PSI exhibited a higher posterior acromion and reduced posterior acromial coverage.^5^

Complementing these anatomical findings, a biomechanical study by Hochreiter et al revealed that the acromion functions as a passive restraint against posterior displacement of the humeral head. Their work showed that a high, flat acromion required 23%-60% less force to displace the humeral head by 50% of the glenoid width, whereas surgical correction to a lower, steeper configuration increased the necessary force by up to 122%.^17^

Furthermore, Akgün et al identified several distinctive acromial features that could serve as predictors for type C1 PSI, including increased anterior acromial coverage (22° ± 6.7° in C1 PSI vs. 14.1° ± 3.4° in healthy controls), reduced posterior coverage (52.8° ± 11.1° vs. 63.2° ± 6.3°), greater posterior acromial height (21.9 mm ± 4.6 mm vs. 18.6 mm ± 4.7 mm), and a steeper posterior acromial tilt (78.8° ± 5° vs. 69.8° ± 3.9°). These structural variations may compromise the acromion's stabilizing role, thereby facilitating excessive posterior displacement of the humeral head under load.^1^

## Scapulothoracic orientation

Proper scapular positioning is fundamental to maintaining optimal shoulder function.^32^ During arm elevation, the scapula follows a coordinated pattern of upward rotation, external rotation, and posterior tilting, which together ensure efficient shoulder motion and joint stability.^26^^,^^43^ Altered scapular kinematics have been linked to various shoulder pathologies, including glenohumeral instability, with studies consistently reporting increased scapular internal rotation, decreased upward rotation,^20^^,^^32^ and altered tilt—specifically, reduced anterior tilt coupled with increased posterior tilt—during arm elevation.^42^^,^^43^ However, data on scapulothoracic orientation in patients with type C1 PSI remain limited.^1^

A study examining scapular positioning in this population found that patients with C1 PSI have significantly reduced scapular upward rotation (9.6° ± 3.6° vs. 12.2° ± 2.4°) and anterior tilt (7.9° ± 3.2° vs. 16.7° ± 3.5°) in the supine static position compared to healthy controls. Although it remains to be fully clarified whether these deviations represent a compensatory response to humeral decentering or contribute directly to its development, scapular malalignment likely leads to biomechanical changes that compromise joint stability and may contribute to shoulder dysfunction.^1^

## Muscle balance

The transverse force couple (TFC) of the rotator cuff, consisting of the anterior subscapularis and the posterior infraspinatus/teres minor, plays a key role in maintaining glenohumeral stability by balancing muscle forces. Disruption of this balance can significantly alter force transmission and load distribution within the shoulder.^36^

Studies in healthy shoulders generally report no significant differences in TFC muscle volume between the anterior and posterior components.^11^^,^^36^ Piepers et al analyzed 27, ^36^ and Espinosa-Uribe et al examined 392 healthy shoulders, noting similar muscle volumes. They also observed that balanced muscle volume generally declines with age as part of a physiological process.^11^

Research on muscle volume in patients with static posterior subluxation is limited.^1^^,^^28^ Mitterer et al evaluated 10 patients with static PSI and found that the subscapularis volume was slightly larger than that of the infraspinatus/teres minor, yielding a volume ratio of 1.14 ± 0.13; however, the lack of a healthy control group in this study limits the interpretation of these findings.^28^ If C1 PSI similarly progresses toward the Walch B–type glenoid, marked by posterior humeral head subluxation and glenoid retroversion, we would expect more marked muscular changes. Indeed, B-type shoulders show increased fatty infiltration of the posterior rotator cuff relative to the subscapularis, leading to TFC imbalances that can disrupt the posterior cuff's length–tension relationship and promote further instability.^3^^,^^47^

However, when focusing specifically on type C1 PSI, Akgün et al compared TFC muscle volumes between C1 PSI patients and an age-matched healthy cohort, finding only a slight difference in volume ratios (1.1 ± 0.2 in C1 PSI vs. 1.07 ± 0.1 in controls). This indicates that the relative muscle volumes of the TFC remain similar between C1 PSI patients and their matched healthy controls.^1^ Notably, they also observed significant differences in the volumes of the trapezius and deltoid muscles, with the C1 group exhibiting greater volumes. Although the clinical implications and underlying mechanisms remain unclear, these findings suggest that muscle volume changes in C1 PSI may extend beyond the TFC.^1^

Recent findings by Plachel et al show that patients with eccentric glenohumeral OA, defined by posterior humeral head decentering and asymmetric glenoid wear, report higher levels of shoulder-straining activities, particularly combat sports and weightlifting. They attribute the higher incidence of glenohumeral OA to extreme repetitive stress and force-couple imbalances caused by training large muscle groups such as the pectoralis major and deltoid while neglecting the stabilizers, a mechanism that may also underlie the higher incidence of C1 PSI in muscular males.^37^

## Clinical presentation and examination

A thorough patient history, including the onset of symptoms, associative factors, and functional demands, is essential for diagnosing C1 PSI and should be integrated with clinical findings to guide management decisions.^18^ During visual inspection, the examiner should focus on evaluating shoulder contour, abnormal movement patterns, asymmetry, muscle atrophy, scapular dyskinesis, and overall posture. Comparing active and passive range of motion, as well as measuring strength differences between the affected and contralateral shoulders, are critical components of the examination.^34^ Furthermore, patients with advanced C1 PSI can present with marked limitations in glenohumeral mobility. Walch et al showed that these restrictions were most evident in forward active elevation and external rotation. Patients reported a progressive decline in shoulder function, characterized by pain, stiffness, mechanical locking, and sensations of instability.^45^

Several instability tests can help differentiate C1 PSI from other forms of PSI,^34^ including the O'Brien test,^24^ FIRO test (forced internal rotation test),^30^ Show-me test,^30^ Kim test, and Jerk test,^21^ and posterior drawer test.^12^ Notably, Moroder et al found that both the Jerk test and the posterior drawer test, each with a sensitivity of 73%, are particularly reliable for detecting C1 PSI.^30^

## Radiographic assessment

A comprehensive radiographic evaluation is essential for assessing osteoarthritic changes and humeral decentering in patients with C1 PSI. Standard views, including true anteroposterior, axillary, and scapular Y projections, provide valuable initial information, while cross-sectional imaging plays a critical role in the diagnosis.^30^

## Glenoid morphology

**Figure 1:**
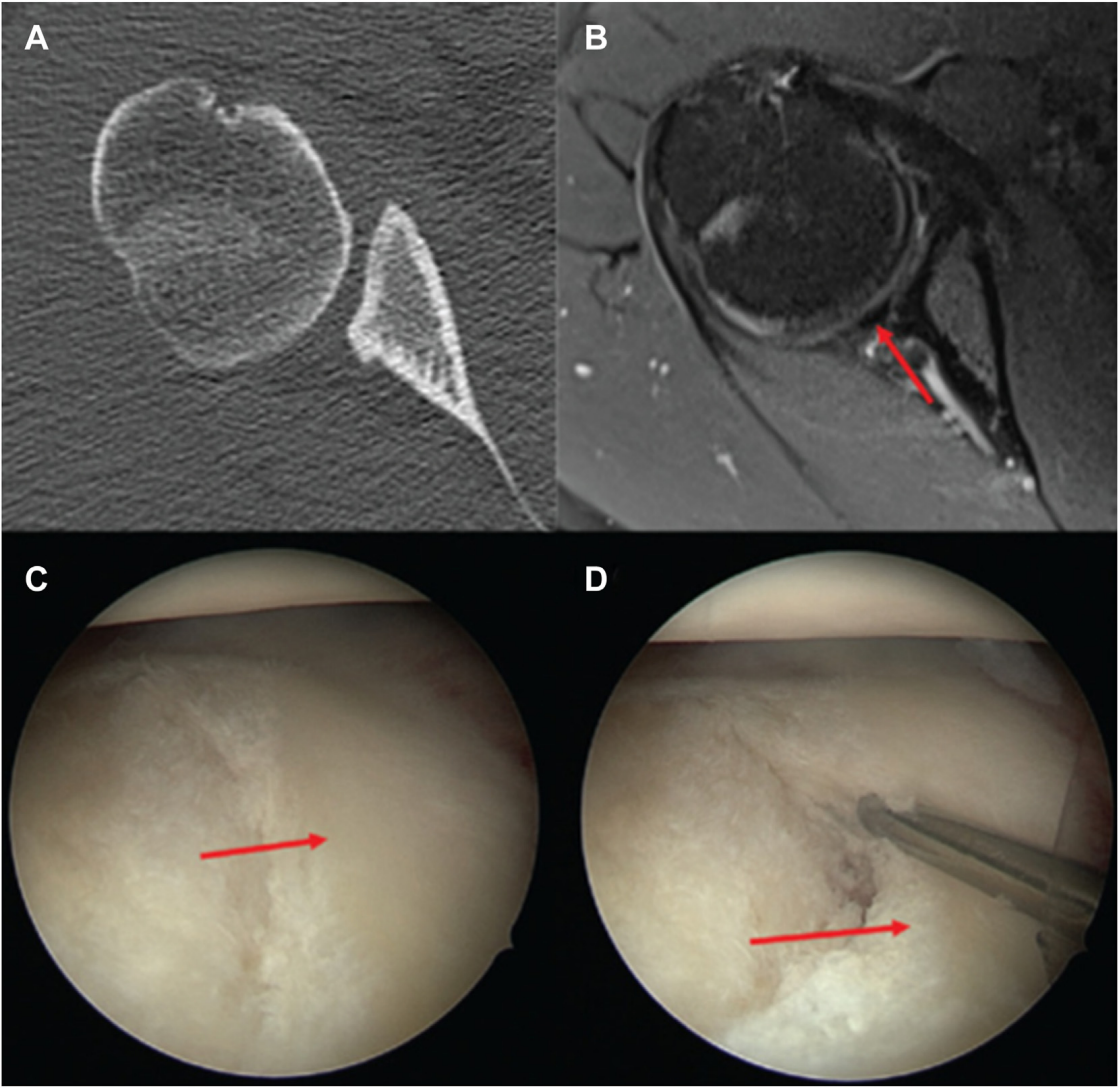
(**A**) Axial CT demonstrating marked glenoid retroversion and a deficiency of the posterior glenoid rim. (**B**) MRI scan revealing a compensatory hypertrophy of the posterior labrum (*red arrow*). (**C** and **D**) Arthroscopic views of a right shoulder with C1 PSI showing pronounced glenoid retroversion and loss of posterior bony concavity, which is offset by a hypertrophic posterior labrum (*red arrows*). Figure reused with permission from Moroder et al^29^. *CT*, computed tomography; *MRI*, magnetic resonance imaging; *PSI*, posterior shoulder instability.

In particular, CT scans may be helpful in assessing humeral centering and identifying bony changes such as increased glenoid retroversion, anterior offset, and posterior glenoid deficiency in the context of glenoid dysplasia (Fig. 1).^30^ Glenoid dysplasia, characterized by a deficient posterior rim, altered mechanical alignment with increased retroversion, and hyperplasia of the posterior labrum,^8^^,^^49^ appears to be common among C1 PSI patients.^1^ Weishaupt et al described 2 forms of glenoid dysplasia in patients with atraumatic, recurrent posterior instability: the rounded “lazy J″ form and the triangular “delta” form.^49^ Furthermore, Akgün et al reported that 29% of their patient cohort with C1 PSI had glenoid dysplasia manifested as the "lazy J sign."^29^

## Acromial morphology

Evaluation of acromial morphology also plays an important role in C1 PSI, as patients often have decreased posterior acromial coverage, increased posterior acromial height, and greater posterior acromial tilt compared to healthy controls.^1^ Y-view radiographs^27^ and 3D reconstructions from CT scans^1^ have proven effective for evaluating acromial angles. In particular, 3D models derived from true sagittal views, where the coracoid process and scapular spine form the symmetrical upper limbs of a “Y,” allow for a detailed assessment of posterior acromial tilt, height, and coverage.^1^^,^^27^

## Posterior labral changes

In C1 PSI, magnetic resonance imaging should focus on identifying posterior labral changes. Moroder et al noted that patients with a deficient bony posterior glenoid rim often demonstrate a hypertrophic posterior labrum, which appear to compensate for structural deficiencies by enhancing glenoid concavity (Fig. 1).^29^

## Glenohumeral relationship and subluxation indices

**Figure 2:**
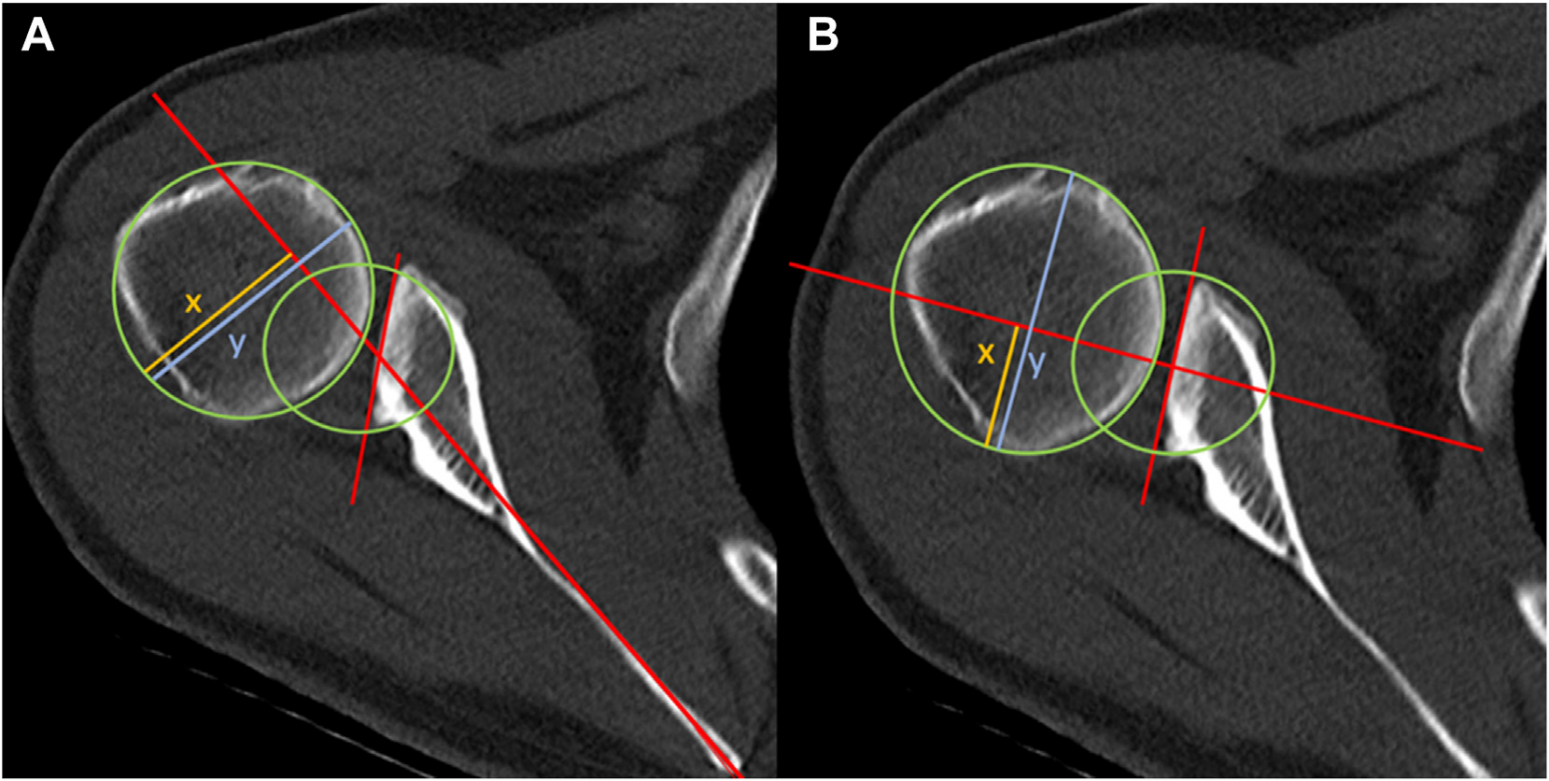
Measurement of the scapulohumeral and glenohumeral subluxation indices on CT of a left shoulder in a patient with C1 PSI. (**A**) The scapulohumeral subluxation index is determined by dividing the length of line x by the length of line y, using the Friedmann line as a reference. (**B**) The glenohumeral subluxation index is measured in the same manner, using the midpoint of the glenoid as a reference. Notably, this figure illustrates the higher scapulohumeral subluxation index in C1 PSI but a glenohumeral-centered joint. *PSI*, posterior shoulder instability; *CT*, computed tomography.

In C1 PSI, both the glenohumeral subluxation index and the scapulohumeral subluxation index can be used to assess humeral subluxation. The glenohumeral subluxation index quantifies the position of the humeral head relative to the midpoint of the glenoid (Fig. 2),^35^^,^^46^ whereas the scapulohumeral subluxation index uses the Friedman line as a reference (Fig. 2).^46^ Although C1 patients show a scapulohumeral decentered joint, the glenohumeral joint remains fairly well centered due to potential compensatory mechanism between humeral torsion glenoid offset, and version (Fig. 2). Accordingly, because C1 PSI is characterized by scapulohumeral rather than glenohumeral displacement, the scapulohumeral subluxation index more precisely quantifies posterior subluxation in this setting.^1^ A scapulohumeral subluxation index of 0.61 or greater may be used as the cutoff for defining posterior subluxation, effectively distinguishing group C in the ABC classification.^30^

## Treatment options

The management of type C1 PSI remains challenging. To date, neither conservative nor surgical interventions have been shown to provide durable benefits or to halt the progression of C1 PSI; nonetheless, a noninvasive conservative approach remains the first-line recommendation. In young, active patients, joint-preserving strategies are preferred.^29^^,^^39^ Surgical options range from soft tissue procedures,^29^^,^^39^ to structural interventions such as posterior open wedge glenoid osteotomy (POWGO), posterior bone block augmentation, and posterior acromion osteotomy.^10^^,^^15^^,^^22^^,^^34^ Although these approaches may provide symptomatic relief, many techniques have failed to consistently recenter the humeral head, and OA progression continues to be a concern.^22^^,^^29^^,^^33^^,^^48^ The different approaches are outlined in the following section, but no stepwise approach can be recommended as the literature is inconclusive (Table I).

**Table I:** Current surgical treatment options for C1 PSI.

Treatment option	Number of shoulders, n	Latest follow-up, mo	Outcomes
Posterior Articular Coverage and Shift (PACS) procedure^29^	14	24	**Improvement**: Subjective outcome and pain scores
			**Subluxation**: No recentering
Arthroscopic Medial Tenodesis of the Subscapularis Tendon (AMTS) procedure^39^	7	12	**Improvement**: Subjective outcome and pain scores
			**Subluxation**: Decreased
Posterior open wedge glenoid osteotomy (POWGO)^15^^,^^33^^,^^48^	8^15^ 7^48^ 10^33^	92 180 33	**Improvement:**^15^ Subjective outcome and pain scores
			**Subluxation**: 2 out of 4 remained centered
			**Osteoarthritis**: Progression
			**Improvement:**^48^ Subjective outcome and pain scores
			**Subluxation**: No recentering
			**Osteoarthritis**: progression
			**Improvement:**^33^ Subjective outcome and pain scores, range of motion
			**Subluxation**: Increased
Posterior bone block procedure^10^	7	24	**Improvement**: Subjective outcome and pain scores
			**Subluxation**: No recentering
			**Osteoarthritis**: No progression
Scope procedure^13^	1	24	**Improvement:** Subjective outcome and pain scores, range of motion
			**Subluxation**: Recentered joint
			**Osteoarthritis**: No progression

*PSI*, posterior shoulder instability.

## Soft tissue procedures

Soft tissue procedures, such as the Posterior Articular Coverage and Shift (PACS) procedure^29^ and the Arthroscopic Medial Tenodesis of the Subscapularis Tendon (AMTS) procedure,^39^ have been developed to address C1 PSI.

**Figure 3:**
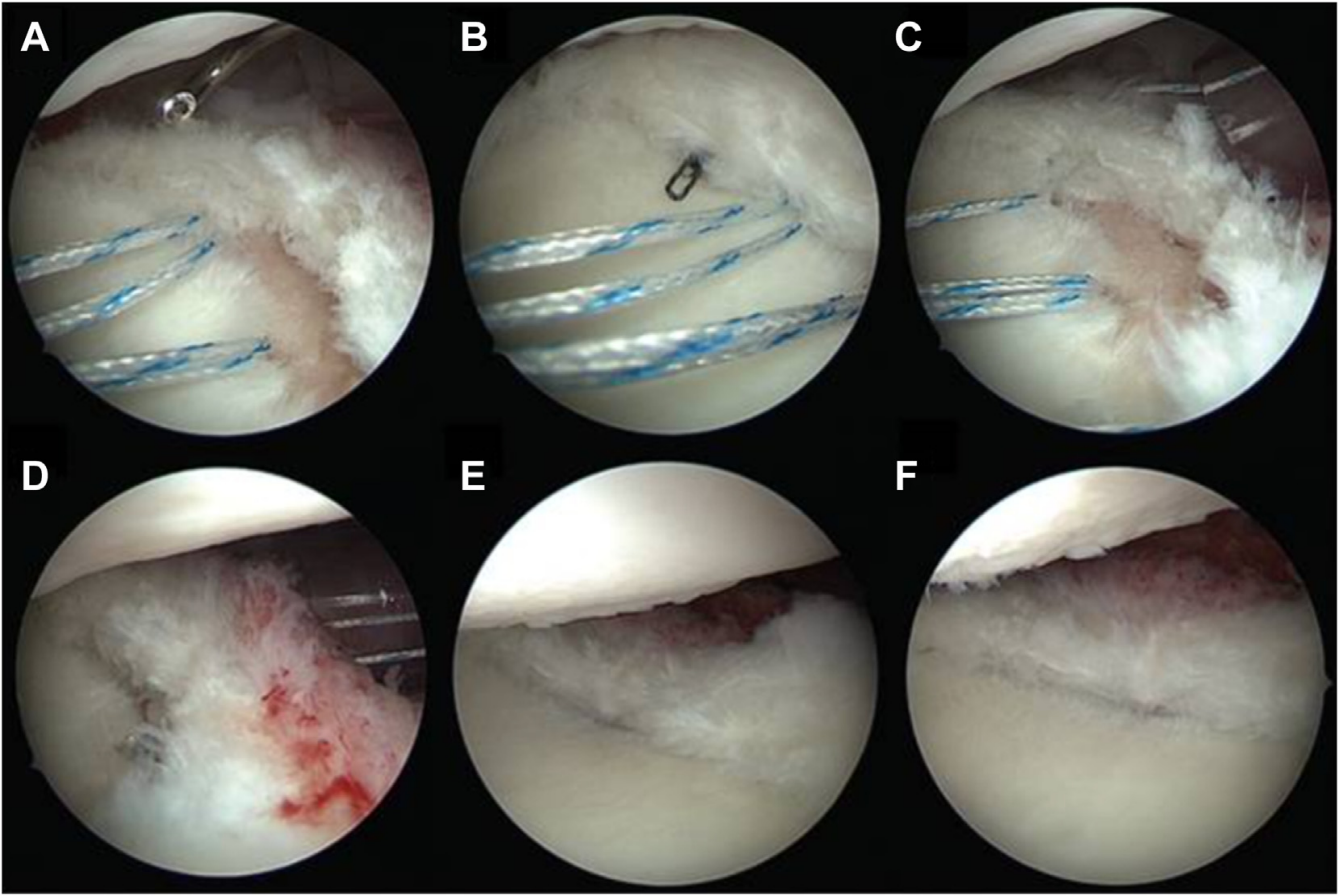
Arthroscopic images from the anterosuperior portal of a right shoulder in lateral decubitus position illustrate the posterior articular coverage and shift procedure. Panels (**A**–**F**) show the suture passage through the posterior capsulolabral complex using a suture lasso, with both suture limbs secured by mattress stitches. The technique reattaches and advances the posterior capsulolabral complex at the junction of native cartilage and defect, effectively covering the posterior cartilage lesion. Figure reused with permission from Moroder et al ^29^.

The PACS procedure involves débridement and microfracturing of the posterior cartilage defect, often observed in C1 PSI, followed by mobilization of the capsulolabral tissue and posterior capsular shift to reattach the posterior capsulolabral complex at the junction between the native cartilage and the defect (Fig. 3). It has been suggested that the hypertrophic posterior labrum may provide a suitable substrate for reconstructing the posterior glenoid rim.^29^

Short-term results have shown symptomatic improvement at two years postoperatively; however, patients with greater glenoid retroversion, a higher preoperative scapulohumeral subluxation index, increased posterior humeral head position, and older age tend to have poorer outcomes. Furthermore, the translational effect on the humeral head appears transient, as immediate postoperative recentering was lost by the 2-year follow-up, with 43% of patients showing progression in OA stage.^29^

The AMTS procedure aims to restore joint biomechanics by altering rotator cuff vectors to counteract posterior subluxation. This technique combines posterior labral repair and glenoid defect filling with the posterior labrum, similar to the PACS procedure. Furthermore, the technique involves medializing the subscapularis tendon. Therefore, a tenodesis of the upper third of the subscapularis is performed with an anchor at a more medial position along the lesser tuberosity. This modification intends to anteriorize the humeral head, thereby promoting dynamic stabilization and recentering of the joint.^39^ In a small case series, the AMTS procedure demonstrated promising results at 1 year, with evidence of humeral head recentering as measured by the scapulohumeral subluxation index.^39^ Nevertheless, results of this procedure may be overstated, as the effect may be due to combined posterior labral repair and may be affected by the loss of reduction seen with that technique.

## Bony procedures

A variety of bony procedures have been developed to address the structural deficits of PSI.^10^^,^^13^^,^^19^^,^^40^ These techniques aim to correct osseous abnormalities such as excessive glenoid retroversion, posterior glenoid deficiency, and altered acromial morphology. Among these, POWGO and posterior bone block procedures focus on optimizing glenoid alignment and concavity,^10^^,^^15^^,^^48^ while acromial osteotomy represents a novel approach aiming to address deficiencies in posterior acromial support.^13^^,^^40^^,^^44^

## Posterior open wedge glenoid osteotomy

POWGO is primarily used in cases with high-grade glenoid retroversion. It involves performing an osteotomy on the posterior scapular neck and inserting a bone autograft,^19^ often following Scott's approach,^41^ with the goal of realigning the glenoid version and restoring stability.^48^

Although some midterm studies report favorable functional outcomes,^15^ residual instability and the OA progression are common.^25^ Hinz et al noted persistent PSI in 75% of patients despite successful retroversion correction,^15^ and long-term data reveal high recurrence rates of instability and failure to recenter the humeral head as measured by the scapulohumeral subluxation index in patients with retroversion greater than 15° after 15 years.^48^ Ortmaier et al demonstrated that while POWGO led to a correction of glenoid retroversion, it did not prevent the progression of posterior humeral head subluxation as indicated glenohumeral subluxation index. They observed that the initial glenohumeral joint recentering achieved through POWGO was lost, and a spontaneous decentering of the humeral head had occurred at the follow-up 33 months after intervention.^33^ These limitations underscore that POWGO should be approached with caution and mainly reserved as a salvage procedure.^22^

## Posterior bone block procedures

Posterior bone block procedures are used to address posterior glenoid deficiency or failed soft tissue repairs, aiming to increase the articulating surface of the posteroinferior glenoid by adding a bone block.^10^^,^^50^

**Figure 4:**
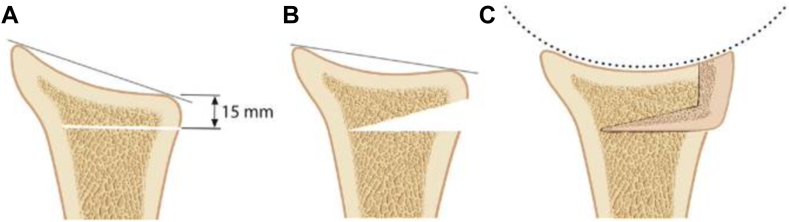
Posterior J-graft iliac crest bone graft technique. (**A**) An osteotomy is performed from posterior to anterior, 15 mm medial to the glenoid. (**B**) The osteotomy is then carefully expanded to create sufficient space for the graft. (**C**) The J-shaped graft is press-fitted into position to restore glenoid concavity and compensate for the posteroinferior deficiency. Figure reused with permission from Ernstbrunner et al^9^.

A biomechanical study combining POWGO with a J-shaped iliac crest bone graft showed that shoulder stability can be restored and glenohumeral contact patterns can be normalized, comparable to intact joints, particularly at 0° of retroversion. Also, less posterior humeral head translation compared to shoulders with 20° retroversion was observed after this procedure (Fig. 4).^9^^,^^14^ In a clinical study of 7 patients with glenoid retroversion ≥15° and posteroinferior dysplasia, J-bone graft union was achieved in all cases, with a 20% increase in glenoid surface area and improvements in stability and pain relief. It is important to note that despite the overall good results of the current study, only 4 of the 7 patients treated had preoperative humeral head subluxation. After the POWGO procedure, only 2 of the patients with posterior humeral head subluxation achieved recentering of the humeral head. Notably, these 2 patients, who were significantly older, failed to demonstrate persistent recentering at final follow-up, developed advanced dislocation arthropathy, and continued to have persistent pain.^10^ While short-term results^10^ and favorable biomechanical outcomes^9^ of this technique seem promising, long-term data especially, regarding posterior humeral head recentering, are lacking to prove whether these results are superior to other approaches.

## Acromial osteotomy

Acromial osteotomy, as first described by Gerber et al, may offer an alternative approach to addressing static posterior subluxation of the glenohumeral joint.^13^ A recent case report on the "SCOPE" procedure, which combines glenoid and acromion osteotomy, demonstrated effective correction of anatomical anomalies associated with static posterior subluxation, resulting in a pain-free, functionally normal shoulder without radiographic OA progression at 24 months.^13^ Despite these encouraging early findings, it is important to note that they are based on a single case report. To confirm the long-term efficacy and generalizability of this procedure, studies with larger patient cohorts and longer follow-up are needed. Moreover, it is important to determine whether it is the combination of glenoid osteotomy and acromion osteotomy or the acromion osteotomy alone that helps recentering the joint.

## Conclusion

Type C1 PSI is a multifactorial preosteoarthritic condition characterized by increased glenoid retroversion, decreased humeral retrotorsion, altered scapulothoracic alignment, abnormal acromial morphology, and muscle imbalances. These factors predispose young patients to chronic posterior subluxation and joint degeneration. Thorough clinical evaluation, including medical history, physical examination, and instability tests, aids early detection, while CT and magnetic resonance imaging scans can identify key features such as glenoid dysplasia, posterior humeral head subluxation, and labral hypertrophy. Various approaches can be used to treat the pathology. These range from conservative therapy and arthroscopic surgery to more open surgical procedures and corrective osteotomies. However, there is no recommended technique or treatment algorithm for C1 PSI, as none of the techniques has been shown to successfully demonstrate humeral head realignment and OA prevention in the long term.

## Disclaimers:

Funding: No funding was disclosed by the authors.

Conflicts of interest: The authors, their immediate families, and any research foundation with which they are affiliated have not received any financial payments or other benefits from any commercial entity related to the subject of this article.
